# Balancing between extremes—Work in hospital‐at‐home

**DOI:** 10.1002/nop2.402

**Published:** 2019-10-22

**Authors:** Heli Vaartio‐Rajalin, Kasanga Ngoni, Lisbeth Fagerström

**Affiliations:** ^1^ Faculty of Education and Welfare Studies Åbo Akademi University Vasa Finland; ^2^ Bachelor of Healthcare Nursing Novia University of Applied Sciences Åbo Finland; ^3^ Nursing@Home & Pal@Home Guy's and St Thomas' NHS Foundation Trust Elmcourt Health Centre London UK; ^4^ Faculty of Health and Social Sciences University of South‐Eastern Norway Kongsberg Norway

**Keywords:** experiences, hospital‐at‐home, interprofessional, nurses, nursing, nursing practice

## Abstract

**Aim:**

To describe HAH staff's perceptions about HAH care, including work structures, processes and outcomes.

**Design:**

Cross‐sectional descriptive study of three HAH units in Finland.

**Methods:**

Three focus group interviews of interprofessional staff members (*N* = 24) were analysed through thematic content analysis (COREQ). In addition, an audit visit was conducted at Guy's and St Thomas' @home service, the United Kingdom.

**Results:**

The Finnish HAH staff perceived they were balancing between different extremes: the patient's and his/her near‐one's opinions and wishes, well‐being and integrity, the promotion of person‐centred care and own work safety, a deeper meaning for work and the need for further support. Both in Finland and the UK, patients were perceived to be satisfied with care and HAH was perceived to save hospital bed places.

## 1 INTRODUCTION

The boundaries between acute, primary, special and social care services are weakening. Professional care at home services have increased significantly in Europe, North America and other parts of the world, with the objectives to reduce healthcare costs (Rostgaard & Szebehely, 2012) and improve patients' possibilities to stay at home while receiving quality care in safe circumstances (Vaartio‐Rajalin, Fagerström, & Santamäki‐Fischer, 2019).

Hospital‐at‐home (HAH) is a service form located on the interface between hospital care and home care. HAH includes the provision of elements of medical care, health care and nursing care normally provided to hospital inpatients, realized in a home setting. HAH is provided to individuals of all ages with acute or chronic, somatic or mental health problems who do not need continuous observation and even individuals in need of palliative care (Bäcklund, Cannerfelt, & Sandlund, 2013). Moreover, through HAH, professional support is provided not only to HAH patients but also patients' near‐ones as co‐clients (Ewing, Austin, Diffin, & Grande, 2015; Vaartio‐Rajalin et al., 2019). Without the inclusion of patients' near‐ones as co‐clients, for those with functional limitations HAH is essentially impossible to provide (Landers et al., 2016).

Societal changes are placing new demands on social and healthcare service infrastructures, processes and outcomes, for example professional competence, interprofessional collaboration and person‐centredness in care. Few studies that focus on HAH staff's perceptions on work in HAH are seen. The aim of this study was to describe how interprofessional HAH staff perceive their work in HAH, including work structures, processes and outcomes.

## 2 BACKGROUND

Similar to North America and other parts of Europe, home care in the Nordic countries has undergone a transition during the past few decades and a new range of terminology has emerged. Home care or home care services can be defined as professional care provided at home to patients with formally assessed needs, including domestic aid offered by social care, primary and rehabilitative nursing care, as well as respite care provided to informal caregivers (Genet et al., 2011). Home health care encompasses a range of activities, from preventive health work to palliative care, all with the goal of enhancing patients' functional health status and quality of life (Brazil et al., 2012). Nowadays, home health care is even offered cumulatively and as part of primary health care: to pertinent working age individuals, individuals with mental health problems, families with ill children and terminal phase patients (Ministry of Social Affairs & Health, 2017). Furthermore, the emergence of the trend towards hospital avoidance has led to the development of care being provided through hospital‐at‐home (hospital‐in‐the‐home, patient‐centred medical home) services, with the acronym HAH hereafter used to refer to such care services.

Hospital‐at‐home is a service alternative usually discussed in relation to hospital avoidance or early patient discharge from hospital care, because one objective underlying the increase in HAH is the reduction in healthcare costs (Toofany, 2008). HAH can be offered as form of primary, specialized or private care or as a combination of these. In one scoping review (Vaartio‐Rajalin & Fagerström, 2019, *N* = 35), researchers found that before a planned hospital stay or in conjunction with hospital discharge, a physician or a rapid response team should conduct a holistic assessment of a patient's physical and psychological health, acute and chronic symptoms, symptom distress, functional status, disease stage, comorbidities and motivation. This assessment could be performed at the hospital, during a proactive or acute, unscheduled home visit or by telephone call. At a minimum, subsequent to a patient giving his/her informed consent to receive HAH, the preparedness of the patient's near‐ones to shoulder some responsibility for the patient's care should be explored. In an interview study of HAH patients and their near‐ones (*N* = 45), researchers found that patients' near‐ones may be elderly, have some health problems themselves or experience altered social roles stemming from HAH (Vaartio‐Rajalin et al., 2019) being too burdening (Farina, 2001). The broadness and depth of this assessment should, of course, correspond to the reason underlying a patient's need for HAH, including anticipated length of care.

To ensure that patients' needs are met, a comprehensive professional competence framework that incorporates the coordination of multiple systems and intra‐ and interprofessional collaboration is needed (Larsen, Broberger, & Petersson, 2017). Following an initial pre‐admission assessment, for HAH staff HAH care includes planning, coordinating, implementing and evaluating advanced care, that is the monitoring of medicine compliance and patients' clinical condition (Ministry of Social Affairs & Health, 2017), taking blood samples and other measurements, giving different infusions and transfusions, intravenous injections or respiratory treatments (Bäcklund et al., 2013). Furthermore, HAH care includes informing, educating, coaching and supporting patients as well as their near‐ones in home care activities and in adapting to role and relationship transitions. HAH is realized in interprofessional teams (comprised of a physician/geriatrician and a nurse; possibly even social workers, pharmacists, nutrition therapists or physiotherapists) together with patient's near‐ones. In regard to the nursing being provided, HAH activities are realized by either registered nurses (RNs), district nurses (DNs) or advanced practitioner nurses (APNs) (Vaartio‐Rajalin & Fagerström, 2019.).

There are some profession‐specific competency requirements for HAH staff. In a scoping review, researchers found that the competence framework for rapid response team members was comprised of 2–30 years clinical experience in a specialty, the undertaking of a physical assessment, the completion of a clinical reasoning course at degree or Master's level, the completion of non‐medical prescribing studies, having a clinical supervisor and engaging in self‐reflection through the use of a competency workbook (Vaartio‐Rajalin & Fagerström, 2019). Other researchers have seen that the competence framework for those engaged in rapid response team work but not nursing per se included employment in senior roles in acute hospital settings*,* specialization or experience in acute care, oncology or gerontology, having or working towards a Master's level degree in advanced practice and/or having completed modules in advanced physical assessment skills and having completed a non‐medical prescribers course (Öhlen, Forsberg, & Broberg, 2013). Also presupposed were, for example psychosocial (Pusa, Hägglund, Nilsson, & Sundin, 2015), communicative, cooperative (Bäcklund et al., 2013), technology, evidence‐based and documentation competencies (Öhlen et al., 2013) and leadership competencies (Lagerstedt, 2012). There are no specific competency requirements in Finland for staff working in HAH other than the requirement to hold a Bachelor of Health Care, Nursing degree (210 ECTS), which results in qualification as an RN. Still, the National Supervisory Authority for Welfare and Health and the Regional State Administrative Agencies monitor all healthcare services and healthcare organization employers are required to check that all employees have relevant qualifications and the professional competencies needed for the tasks they will perform, especially in relation to medication and device security. The National Institute for Health and Welfare can also set additional competence requirements for employees in relation to a given work place. In an interview study of HAH patients and their near‐ones (Vaartio‐Rajalin et al., 2019), researchers saw that the participants (*N* = 45) perceived the important characteristics of HAH nurses to include not only professional clinical competence but also service‐mindedness and flexibility, as well as respect for the patient's situation, home and right to participate in decision‐making.

Advanced practitioner nurses are often responsible for clinical history taking, drawing up care plans and coordinating care teams (Fagerström, 2010). In the home care context, APNs are known to shoulder great responsibility for the advanced health and nursing care being received and researchers have demonstrated that APN‐led care results in equivalent or better outcomes than physician‐led services in regard to the reduction in *s*ymptom burden, self‐management and behavioural outcomes, disease‐specific indicators, patient's satisfaction and perception of quality of life and health service use (Chan et al., 2018; Pouliot, Weiss, Pratt, & DiSorbo, 2017; Vaartio‐Rajalin & Fagerström, 2019). The ICN Nurse Practitioner/ Advanced Practice Nursing Network (https://international.aanp.org/Practice/APNRoles) defines an APN as, “a registered nurse who has acquired the expert knowledge base, complex decision‐making skills and clinical competencies for expanded practice, the characteristics of which are shaped by the context and/or country where s/he is credentialed to practise.” Compared with specialist nurses, APNs generally have a 2‐year Masters level degree and more advanced skills in advanced nursing and medical care and, usually, the extended authority to prescribe medication. APNs are also to some extent involved in research assistant duties such as enrolling study subjects, abstracting medical records, collating study materials or disseminating completed study results, which are important when developing and evaluating evidence‐based care structures and processes.

While clinical symptoms primarily guide the decision‐making process connected to HAH planning, implementation and evaluation, the “whole patient,” seen as a psychosocial and existential person, must be taken into consideration during care at home. The patient's near‐ones should also be engaged in HAH care‐related processes (Ewing et al., 2015), because a patient's near‐ones can experience different role and relationship transitions stemming from HAH (Vaartio‐Rajalin et al., 2019). Such patient and near‐one engagement is one dimension of person‐centred care, which has been defined as:

education and shared knowledge in terms of timely and complete information on patient prognosis, progress and disease process; appropriate involvement of family and friends in decision‐making and information giving; the sense of inter‐provider collaboration and team management; sensitivity to non medical and spiritual dimensions of care; and respect for patient needs and preferences in care. (Shaller, 2007)

Person‐centred care has also been defined as respect for the personal narratives that reflect a person's sense of self, lived experiences and relationships and the recognition of this respect through the safeguarding of a partnership in shared decision‐making and in meaningful activities in a personalized environment (Ekman et al., 2011; Kitwood, 1997; McCormack & McCance, 2006). In a scoping review with an HAH context (Vaartio‐Rajalin & Fagerström, 2019), researchers found that patient‐centredness was perceived as respect for a patient's autonomy, self‐determination capacity and social relationships and made concrete through a continuous, trustful relationship established during the planning and evaluation of care by nurses together with the patient and his/her near‐ones. Patient‐centredness was thus based on the patient's needs while still being financially viable, with care taking place in the patient's home (seen as the patient's “own”) environment. In that review, APNs, DNs and/or RNs were considered an instrumental factor in the facilitation of patient‐centredness (Jeangsawang, Malathum, Panpakdee, Brooten, & Nityasuddhi, 2012; Ljungbeck & Sjögren‐Forss, 2017; Pusa et al., 2015). In other research on HAH outcomes, patients and their near‐ones were seen to have experienced safety, satisfaction, reduced clinical symptoms and better physical, mental and social functionality due to enhanced choice and support from the team providing home care (Vaartio‐Rajalin & Fagerström, 2019; Vaartio‐Rajalin et al., 2019). The aim of this study was to describe how interprofessional HAH staff perceive HAH care, including work structures, processes and outcomes. The research questions concerned HAH staff's perceptions of HAH, including work structures, processes and outcomes:

- How are the patient and his/her near‐ones taken into consideration both before and during the HAH care process?
- What all does HAH care involve and how does the patient's home affect the HAH care process?
- Which professional competencies are relevant and what does interprofessionality mean in the HAH context?
- What is the effect of HAH care?

## 3 DESIGN

This was a cross‐sectional descriptive study of three HAH units in Finland. The strategic sample included two HAH units offering services during the day and evenings on weekdays and weekends (Units A and B) and one unit offering services during the day, evenings and overnight on weekdays and weekends (Unit C). Unit C falls administratively under specialized health and nursing care, while Units A and B fall administratively under primary health care.

## 4 METHODS AND ETHICS

Subsequent to approval from each participating organization's ethical committee, the charge nurses for the HAH units included in this study recruited voluntary HAH nurses and physicians using a researcher‐developed information sheet. Written informed consent was considered a sign of voluntariness. The information sheet contained information about the aim of the study, data collection procedures, participants' right to self‐determination and aspects of confidentiality and anonymity. Participants also received information about who to contact and how for additional information (see Consolidated criteria for reporting qualitative studies (COREQ, Appendix S1).

The research questions, definitions of person‐centredness and the central attributes of the HAH context formed the foundation from which the interview questions (Table 1) were developed. The order of the questions was flexible and depended on the participants' answers.

**Table 1:** The interview questions

How would you describe the process through which the patient is admitted to HAH care? How do you take into consideration the patient and his/her near‐ones when planning HAH care? How do you obtain their informed consent? What does HAH care include, what all does it involve? How does the patient's home affect HAH care? What does interprofessionality mean in the HAH context? Who shoulders the main responsibility for the patient care and rehabilitation? Who coordinates the patient care? Who leads the team? Who does what? Who evaluates the care? Which competencies do you perceive are relevant for a person qualified to work in HAH care? How do you perceive that HAH affects… …the patient? …the patient's near‐ones? …the HAH staff? …society? In what ways and by whom is the patient care received through HAH evaluated?

During spring 2019 (February 28–April 10), focus group interviews of mixed groups of HAH nurses and physicians were conducted. In addition to interview questions, some background variables (age, gender, HAH work experience, care work experience, official professional title) were collected at the beginning of the interview.

In addition to focus group interviews, an audit visit was conducted in May 2019 at Guy's and St Thomas' @home (GSTT@home) service in London, the UK. The purpose of the visit was to reveal the differences and similarities between HAH care in Finland and the UK. During the visit, an individual interview with the Deputy Head for the service, using the same interview questions as used during the focus group interviews in Finland, was conducted.

GSTT@home service is a nurse‐led service that provides HAH services for the local communities in the London boroughs of Lambeth and Southwark, which together comprise a highly diverse resident population of approximately 600,000 where over 300 languages are spoken. The catchment area of this HAH service includes two major teaching hospitals (Guy's and St Thomas' and King's College Hospital).

## 5 ANALYSIS

The data were tape‐recorded and analysed through inductive thematic content analysis (Elo et al., 2014) with a focus on manifest content. As units of analysis, both sentences and parts of the text that represented the idea underlying the whole were applied (Table 2).

**Table 2:** Examples of content analysis

Codes	Subcategories	Category	Theme
The patient is asked how he/she manages at home The patient is asked how he/she perceives he/she currently manages at home	The patient is asked how he/she perceives he/she currently manages at home		
The patient is asked whether he/she has previously used home care services We ask about his/her near‐ones and whether they can assist him/her at home	The patient is asked about near‐ones and previously used service forms when HAH care is considered	When HAH care is considered, the patient and near‐ones participate in the decision‐making	Pre‐admission to HAH: Balancing between the patient's and his/her near‐ones' opinions and wishes
The patient is asked where he/she wants to receive care The patient expresses his/her desire to be cared for at home The patient states, “I want to go home.”	The patient is asked where he/she wants care to take place		
The patients' near‐ones are asked whether they accept HAH care prior to the start of care The near‐ones are also asked for their opinions	The patient's near‐ones are asked whether they accept HAH care		

## 6 RESULTS

Altogether three interprofessional teams (*N* = 24, 20 nurses and four physicians, age 26–58, mean 44) with care work experience ranging from 3–30 years (mean 20.8) and HAH work experience ranging from 2.5 months–18 years (mean 6.6) were interviewed. Of the participants, two nurses were enrolled in an APN educational programme, two physicians had no specialization and two were specialized in internal medicine.

In Focus group A, there were four nurses and one physician, age 27–48 years (mean 40), care work experience 5–20 years (mean 11) and HAH work experience 3.5–7 years (mean 4.8). In Focus group B, there were five nurses and one physician, age 37–58 years (mean 50), care work experience 15–30 years (mean 28 years) and HAH work experience 2–3.5 years (mean 2.9). In Focus group C, there were 11 nurses and two physicians, age 26–57 years (mean 43.5), care work experience 3–30 years (mean 17.6) and HAH work experience 2.5 months to 18 years (mean 9.9). The participants' gender and education levels were not reported on the unit level to ensure anonymity.

The focus group interviews took place during working hours, in a meeting room at the relevant HAH units. The interviews lasted between 54–78 min. A total of 58 subcategories, 15 categories and eight themes were identified from the content analysis (Table 3).

**Table 3:** Subcategories, categories and themes

Subcategories	Categories	Themes
The patient is asked how he/she perceives he/she currently manages at home The patient is asked about near‐ones and previously used service forms when HAH care is considered The patient is asked where he/she wants care to take place The patient's near‐ones are asked whether they accept HAH care	When HAH care is considered, the patient and near‐ones participate in the decision‐making	Pre‐admission to HAH: Balancing between the patient's and his/her near‐ones' opinions and wishes
The nurse makes observations during the first home visit The patient and his/her near‐ones are given a chance to reveal narratives The patient's extended family is taken into consideration as being important for the patient The care is flexibly planned in accordance with the patient's situation, needs and preferences The patient is involved in the evaluation and development of care	During HAH care, the patient and his/her near‐ones are focused on	HAH care process: Focusing on both the patient and his/her near‐ones during care
Home seen as a place of equality The patient's and his/her near‐ones' integrity is respected The patient's home is respected Home seen as a resource to understand the patient, his/her background and the actual situation	Home as a source of person‐centred care	Home: Balancing between the promotion of person‐centred care and own work safety
Home milieu seen as a challenge Being responsible for one's own safety Being responsible for one's work conditions	Home as a challenge to staff safety	
Clinical skills Specialized nursing experience Seeing and analysing the whole situation Independent decision‐making	Independent clinical decision‐making on an advanced level	Presupposed competence: Engaging in iterative situation analysis and decision‐making on the individual and team levels
Proactive situation analysis Flexibility Creativity Advocacy skills Continuous learning	Proactive and reactive professional attitude	
Complementary mix of competencies in team Collaboration and communication	Collaboration	
Periodic, acute advanced nursing care Periodic care for long‐term illness, linked to acute care needs Monitoring health and preventing illness Supporting self‐rehabilitation	Tangible work for the HAH patient	Coordinating and developing safe patient care through tangible and intangible measures
Coordinating care for the “whole patient” Being on call Documenting and reporting on patient safety and care continuity Advocating for the patient's best Testing new virtual methods for patient care	Intangible but necessary work for the HAH patient	
Reflecting on and evaluating care Acknowledging the need for nursing advocacy Mentoring nursing students Assisting others staff on other units	Collegial work	
Genuine collaboration between HAH physicians and nurses Collaboration between service units Collaboration with other professionals not belonging to the HAH team	Collaboration between units and professional groups for the patient's best	Collaborating for the patient's best
Patients feel thankful Patients feel safe Patients feel empowered Patients recover sooner than in hospital and live life despite health problems	Patients perceive well‐being despite ill health	Balancing between the patient's well‐being and near‐one's integrity
Near‐ones feel thankful Near‐ones feel relief that care is organized in the home Near‐ones feel burdened Near‐ones experience an intrusion into their private space	Near‐ones have mixed feelings	
Staff perceive a deeper patient–nurse relationship Staff simultaneously experience independence and genuine collaboration when working in HAH when compared with hospital care Staff feel motivated to work Staff acknowledge the effectiveness of their work Staff feel a desire for professional self‐development	Staff perceive that a deeper meaning underlies HAH work	Balancing between a deeper meaning for one's work and the need for further support
Staff feel challenged Staff feel frustrated	Staff perceive a need for support	

As seen in the interview data, certain structures allow HAH staff to take the patient and his/her near‐ones into consideration before and during the care process: when HAH care is considered and when the patient and his/her near‐ones participate in the decision‐making concerning where care should take place. The prospective HAH patients are asked to reflect on their current self‐care capacities at home and their near‐ones and previously used home care services before they are asked where they would like their further care to take place. Also, the patients' near‐ones are asked whether they accept HAH care:

The patient has to manage to cook and perform daily activities by him/herself, or have someone else to take care of all daily tasks such as cooking, hygiene and so on – not always a near‐one, but home service, for example ‐ otherwise he/she cannot become our patient…. The patient is asked during the first evaluation visit upon referral to [HAH services] how he/she manages at home, about his/her perception about their self‐care capacity and suitability of their home for HAH (Focus group C)

I [as a physician] nearly always call the patient's near‐one when we plan HAH care, to inform and to discuss… So that the idea gets their acceptance. And the nurses from the health care center and from the hospital usually contact the near‐ones before the patient is given a referral to HAH care, during hospital discharge… (Focus group A)

The pre‐admission phase to HAH care can be described as balancing between the patient's and his/her near‐ones' opinions and wishes. The HAH care process, then, was perceived to include focusing on both the patient and his/her near‐ones. During an initial home visit, an HAH nurse makes observations about the patient's situation, resources and home as the context for care. The patient and his/her near‐ones are given the chance to reveal their narratives, the patient's extended family (e.g. children, grandchildren, pets) is taken into consideration as being important, the care is planned flexibly in accordance with the patient's situation, needs and preferences, and the patient (but not near‐ones) is involved in the evaluation and development of care:

During the first actual visit to the patient's home you really see the whole picture: their home, their functional capacity, have they understood what HAH care means… The truth can be opposite of what is said… (Focus group C)

The home context makes the care relationship different from the hospital ward, the home makes the patients equal with us nurses… less hierarchical… The care is given based on patient's terms… (Focus group C)

We take the patient's near‐ones and dogs or cats into account, we show our respect for them by saying some words to near‐ones or clapping the pet… Or by taking the blood pressure also of the spouse, if they so wish. (Focus group C)

Palliative patients' home visits take a longer time than other [visits]… There is the whole situation to be taken care of, the patient's near‐ones with their anxieties and worries, they must be given a chance to “unload”. (Focus group B)

We have a care evaluation or satisfaction form, which is given to the patient during the last home visit…They are summarized during unit meetings and used as a basis for quality development. (Focus group C)

Hospital‐at‐home staff perceived that the patient's home influenced HAH care and was a source of person‐centred care. The home was considered a place of equality, a place where the patient's integrity and his/her near‐ones' integrity are respected and the home itself is respected and seen as a resource to understand the patient, his/her background and the actual situation. The HAH staff also perceived that the home was a challenge to staff safety and taking care of one's own safety and work conditions were noted. The home was perceived as balancing between the promotion of person‐centred care and own work safety:

When you enter the patient's home, you are a guest… someone going in with his/her permission…You cannot take decisions by yourself. (Focus group C)

During a home visit there is only the patient and the near‐one(s), no alarm calls from other patient rooms at the same time… You can fully concentrate on this specific patient and this situation and give your full attention… We don't have work uniforms with a large health and nursing care logo, because we want to maintain the patient's privacy while going in and out from his/her house… Same with the cars, we don't announce that we are from HAH… (Focus group C)

You must look at the patient's home to understand his/her situation and resources and maybe you first then understand the whole situation and the reason for his/her health problems… If the patient fails to agree to let us HAH nurses into his/her homes, there might be socioeconomic problems like alcoholism, drugs, social problems, poor social relationships… (Focus group B)

Sometimes it is really difficult to identify the actual address, where the patient is said to live… Or it is a tall building without an elevator, and you must carry up all your supplies… And during the wintertime when we can have up to 80 cm snow, you can't get near the building with your car… and all the fluids you have with you are too cold to be given immediately… (Focus group C)

Our ergonomics is harder to maintain at the patient's home than in hospital, there can be very limited space and the level of hygiene can often be demanding at home…and there might be pets disturbing wound care or IV antibiotics. (Focus group A)

We don't admit patients with alcohol or drug problems to HAH… They are untrustworthy when it comes to being at home [at a certain agreed‐upon time] and they usually have many friends under the influence of alcohol or drugs at home, too, which makes the care situation very unsafe… (Focus group B)

Certain professional competencies related to the fluctuating and sometimes acute nature of HAH care were identified. The participants emphasized the need for clinical skills, specialized nursing experience, seeing and analysing the whole patient situation and independent decision‐making, all connected to independent clinical decision‐making on the advanced level. They also stressed a proactive situation analysis, flexibility, creativity, advocacy skills and continuous learning, seen as a proactive and reactive professional attitude. Furthermore, a complementary mix of competencies in a team and collaboration and communication were emphasized. This was seen as presupposed competence: engaging in iterative situation analysis and decision‐making on the individual and team levels:

Intravenous medication and nutrition, not only periphery cannula but central venous cannula, pain medication, VAC care… taking blood samples… knowing and understanding possible complications and signs of complications… And even more in the future, skills in palliative care are expected… It is important to have work experience from different clinical contexts and from acute situations. (Focus group C)

If we have some nurse working some shifts for HAH instead of working on the ward, they usually don't see the whole patient situation and coordinate his/her holistic care, they only perform the necessary interventions… It is the invisible work which guarantees patient safety, care quality and care continuity. You are alone there during the home visit and make decisions alone… of course you can call your colleague, but it is really a huge responsibility we carry… It is not seen in our wage, in any way…There have been situations where we have saved the patient's life, it [does] not only [occur] in the ER or ICU…. (Focus group A)

I see my role of [being] a physician as more like that of a consultant… the nurses discuss with me whether there is need to move the patient from HAH to hospital, or only to take some radiological analyses, blood samples… They make the clinical observations and situation analysis, I never visit the patient at home. (Focus group B)

We strive to be proactive, draw up instructions in advance before weekends that if the patient's blood sample answers are like this, increase the dosage like that… (Focus group C)

Sometimes one has to point out to the acute care physician or surgeon [such as] this patient cannot be taken care of through HAH, I have worked as a nurse so long that I can see that already… (Focus group B)

It would be useful to have this restricted right for nurses to prescribe some medication, often during weekends, it would be so easy to start a [course of] antibiotics against a urinary tract infection – which is so easy to diagnose – and not to have to wait until Monday (Focus group A)

The processes involved in HAH care were perceived as coordinating and developing safe patient care through tangible and intangible measures. The tangible work that was coordinated for HAH patients included periodic, acute advanced nursing care, periodic care for long‐term illness linked to acute care needs, the monitoring of health and preventing of illness and the supporting of self‐rehabilitation. The intangible but necessary work for the HAH patient consisted of coordinating care for the “whole patient,” being on call, documenting and reporting on patient safety and care continuity, advocating for the patient's best and testing new virtual methods for HAH care. The participants also engaged in collegial work by reflecting on and evaluating care, acknowledging the need for nursing advocacy, mentoring nursing students and assisting in units:

Intravenous antibiotics, intravenous nutrition or PEG… blood transfusions… other intravenous medication such as bone medication, iron medication…. Large wound requiring care 6 times a day, VAC wound care, stoma care in the beginning when the patient is not used to managing the stoma him/herself and needs education and support… fistula care… During summer time often burns… Balancing diabetes patients' glucose level… home dialysis….If we would not exist, the patient would be an inpatient in the hospital. We have tried video visits, too, but there have been some technical problems… and we are going to start testing distance stethoscopes again… and we have a new pain medication pump system with distance monitoring possibilities, how many PCA doses the patient has used… (Focus group C)

Many of our patients have also illnesses other than the acute one, their earlier symptoms can be worse… Often we notice that the earlier illnesses such as hypertension have become more serious due to the acute illness, or associated care… So their earlier medication has to be changed because of the acute illness and its care…and we take care of the whole situation… Documentation is very important … for patient safety and care continuity and collaboration… it takes a lot of time, the structured documentation system and in addition we call each other quite often. (Focus group A)

It is embedded in advanced care that one always encourages patients to test their limits in physical activities… (Focus group C)

It is so much more than to go into a patient's home and perform a task and come out… It is the whole situation, the whole patient is to be considered and coordinated… and the near‐ones…Before, during and after the actual home visit… To arrange consultations, to order blood samples or medicines… (Focus group B)

There, in their homes, the patients often tell and reveal something they would never mention while on a hospital ward and first then can you pass along their message. (Focus group C)

We have no extra staff, it is just us and we try to substitute for one another but it is not always possible… You instead try to manage the day though you are in fact sick… (Focus group A)

Interprofessionality in HAH was in these data perceived as collaborating for the patient's best. The participants described genuine collaboration between HAH physicians and nurses and collaboration between service units and with other professionals external to the HAH team:

The physician makes a referral, from an acute care unit, or a hospital ward, or from a primary health care center… or elderly care service home… to us, or consults us ‐ our physicians or us nurses ‐ what do we think is this patient suitable for HAH and we go and visit the patient in his/her home. We have a genuine collaboration, we have planned this HAH unit together, nurses and physicians and we often make decisions together… the physician has to rely on the nurses' situation analysis and trust our professional competence when making decisions… (Focus group B)

We collaborate with home service and home health care… palliative outpatient clinic…. We discuss and make decisions such as who takes care of which patients based on patients' situations and resources… And of course, we collaborate with the patient and his/her near‐ones…. (Focus group C)

We collaborate all the time with different physicians like a surgeon, gynecologist… physiotherapist, ergotherapist, oncology nurse, social worker, pharmacist… occupational health care… But they have a different documentation system than we have…. podiatrist, nutrition therapist… priest… mortician… The crisis team, police, guard… (Focus group C)

The care received through HAH was perceived to be related to certain outcomes for patients, patients' near‐ones, HAH staff and, indirectly, society and was perceived as balancing between the patient's well‐being and near‐one's integrity. The HAH patients perceived well‐being despite ill health and were found to feel thankful, feel safe, feel empowered and recover sooner than in hospital and live life despite health problems. Yet the patients' near‐ones were found to have mixed feelings and were perceived to feel thankful, feel content and feel relief that care was organized in the home, but could also feel burdened and experience an intrusion into their private space:

We have very thankful patients and near ones, it is the main thing which helps one to go on…Their respect is more tangible in the home than in the hospital. They prefer that we are the same nurses all the time, not a new nurse every shift and every day… they feel safe. (Focus group A)

In all the care evaluation questionnaires we have collected for 11 years, the patients always mention first that they perceive HAH care to be safe… I had always thought that ICU would be safe, but that HAH care…It could have to do with the home context as such, the safe place. They feel safe even though there is no nurse present [around the clock]… One patient told me that on the ward he had to wait for a nurse for over one hour, but at HAH we always answer the telephone and arrive at [a] home within 20 minutes, if necessary. (Focus group C)

The patients recover much sooner, don't get any bacteria like in the hospital… They rehabilitate themselves merely by doing ADL activities at home, they eat better, sleep better…They enjoy their normal life despite the health problem! They have a lot more social contacts, friends and relatives visit them at home rather than in the hospital…Some of them go to work, visit the theater or cinema, take a trip somewhere… (Focus group C)

The patient's near‐ones feel relief when their loved‐ones are at home and they know they can ask us, call us… But sometimes the near‐ones don't want the patient to receive care at home, because they are so tired of their official caretaker role and prioritize a short period of free time, when the patient is taken to the ward for care. (Focus group C)

The patient's near‐ones can also become quite tired of having us in their homes for a long period of time, it is understandable… It disturbs their private life and private sphere… (Focus group B)

In regard to outcomes on the staff level, the participants perceived they were balancing between a deeper meaning for one's work and the need for further support. The HAH staff were found to perceive that a deeper meaning underlies HAH care and were seen to perceive a deeper patient–nurse relationship, simultaneously experience independence and genuine collaboration in HAH care compared with hospital care, feel motivated to work, acknowledge the effectiveness of their work and feel a desire for professional development. At the same time, they could perceive a need for support and they were seen to feel challenged and frustrated:

This work at HAH is different from ward care because here you have to give something of yourself… it also helps the trust relationship to develop when you tell the patient a little bit about yourself… to be a human being to another human being. (Focus group A)

This is at the same time independent work and collaboration…You always have some back‐up…the physician is always available, you don't need to wait until the next day or next shift. (Focus group C)

HAH does not automatically lead to economic benefit, but it shortens the care periods… There is no need for an isolation room and isolation staff, when we take care of those patients in their own homes. And ward patient [beds] are quite expensive, there not only nursing staff is needed but nutrition, cleaning, washing…If HAH did not exist, there would be many more patients in the hospital and [the hospital doesn't] have the resources to take care of those they have now, either! ….. (Focus group C)

We don't have staff turnover and very few sick leaves though this can also be physically demanding work… We all the time have people wanting to work with us in HAH, never a problem to get substitutes….it also says something about HAH… (Focus group C)

I perceive that I learn all the time about human life more and more…..This can be demanding but at the same time very rewarding… (Focus group C)

This work in HAH is different from OR care, for example, because here the patients and their near‐ones are truly present all the time and have different challenges to meet and you must really think about how you introduce certain issues into discussion…That is really challenging… (Focus group A)

I wish public policy makers would understand the comprehensiveness of our competencies and responsibilities in relation to resource allocation and our wages… Every time some development in HAH is discussed, the foremost principle is person‐centeredness, but when it comes to the realization of that development idea, there is no energy nor money to do anything about these problems hindering person‐centeredness… For example no‐one does anything to coordinate the documentation systems between units! (Focus group C)

These results were compared with an individual interview (55 min) of the Deputy Head at Guy's and St Thomas' @home (GSTT@home) service in London, who has lengthy experience of HAH care. As in Finland, in GSTT@home the two founding objectives are to facilitate early discharge from local hospitals and prevent avoidable hospital admissions by means of person‐centred care based on a clinical review (status and patient's situation). Referrals are taken directly from hospitals and community‐based health practitioners, including London ambulance service, district nurses and general practitioners (GPs). GSTT@home have a 7‐day service which is open including on public holidays. The core operating times are 8 a.m. to 8 p.m. when most visits are carried out by the multi‐disciplinary team. From 8 p.m.–11 p.m., the service has a limited nurse‐only team who are responsible for responding to urgent patient requests and the administration of intravenous medications.

The team consists of a service leader (Master of Nursing Science, MNSc), six matrons with ongoing education at the clinical nurse specialist (CNS) or MNSc level, 32 RNs, 15 nurse assistants, two pharmacists, seven physiotherapists, three occupational therapists, two social workers, 15 administrators and two drivers. The service employs two CNSs as a hospital‐based in‐reach team, and they work closely with ward and accident and emergency teams to identify patients suitable for early discharge. Work rotation on a medical hospital unit or in acute care before HAH employment is highly recommended. Medical expertise is provided through six consultant‐led sessions—provided by a team of five hospital‐based geriatricians who visit the service to lead multi‐disciplinary meetings (MDMs). In addition, GSTT@home have a contract for the provision of three GPs per day from Monday to Friday and two GPs on Saturday and Sunday whose main responsibility is to go on home visits to provide medical expertise for the patients. The GPs also play a role in the education and development of the team in acquiring advanced assessment and prescribing skills among the RNs and therapists. The service has a fleet of 11 pool cars to help with the team's transport needs.

The service has capacity to deliver 64 unique patient contacts each day using the various healthcare professionals. Unique visits are defined as any visit completed by a healthcare professional excluding the nursing assistants. Referrals from most specialties are accepted except for paediatric, psychiatric and gynaecology patients. The main reasons for referrals to the service are heart failure, chronic obstructive pulmonary disease (COPD), pneumonia, cellulitis, urinary tract infections, resolving delirium, dehydration, hyperemesis, medication titration and blood monitoring. Like all NHS services in the UK, GSTT@home is free at the point of access. It is funded by the commissioners for the London boroughs of Lambeth, Southwark and Lewisham.

When the HAH services in Finland and GSTT@home were compared, some similarities and differences between the structures, processes and outcomes were identified. In GSTT@home, both the pre‐admission phase and actual care period seem to include a focus on the patient only. During the referral process and the initial visit to the patient's home, verbal informed consent was sought from the patient but not the patient's near‐ones. The patient was reviewed at least once every day and if required up to three times per day, determined in collaboration with the patient. The main principle of HAH care both in Finland and GSTT@home would appear to be the iterative situation analysis of the patient situation, despite slightly different patient groups. Still, in GSTT@home there is a greater focus on curative clinical interventions and coordination of patient care than the intangible patient care seen in Finland. In both Finland and GSTT@home, HAH care seems to be realized through interprofessional collaboration: in GSTT@home, the care plan is reviewed during MDMs led by consultant geriatricians at least three times per week and the caseload is divided into 2 virtual wards with 3 separate MDMs for each team. Virtual and/or digital devices were not used in patient care. In both Finland and GSTT@home, patients are involved in the evaluation of care, but near‐ones are only involved in HAH care in Finland. In Finland and GSTT@home, the patient's home can be a challenge to staff's work safety; among others, alcohol and drug problems are also prevalent in the GSTT@home setting. GSTT@home staff making home visits wear a safety device that records staff's GPS coordinates and can record sound and/or provide live audial transmission of a visit to staff at a GSTT@home bureau. This allows for the submission of evidence in court, if needed, in cases of violence, etc. Comparisons of patient or staff satisfaction in regard to pre‐admission to HAH care between Finland and GSTT@home cannot be made; due to NHS guidelines, perspective HAH patients, their near‐ones and staff cannot be interviewed about the audit visit. However, according to the Deputy Head of GSTT@home, HAH care in the UK setting seen here has certain outcomes in relation to patients and society. GSTT@home patients are satisfied with their care and on average GSTT@home accepts about 220 new patient episodes each month and delivers at least 2,300 visits to patient homes, saving 42 hospital beds each day, that is reducing pressure at the local hospitals.

## 7 DISCUSSION

The aim of this study was to describe how interprofessional HAH staff perceive HAH care including work structures, processes and outcomes. The results of interprofessional focus group interviews in three units in Finland were compared with one audit visit to an HAH unit in London, the UK, during which the Deputy Head of the unit was interviewed.

From the data, we saw that the facilitation of early discharge from hospital and the prevention of hospital admissions—that is improving patients' possibilities to stay at home while receiving quality care in safe circumstances (c.f. Vaartio‐Rajalin et al., 2019) and reducing healthcare costs (c.f. Rostgaard & Szebehely, 2012; Toofany, 2008)—were the objectives of HAH service. We considered these objectives to have been reached, because in the Finnish data we saw that HAH patients were perceived to be satisfied, feel safe, feel empowered and recover sooner than in hospital and live life despite their health problems when receiving HAH care (c.f. Vaartio‐Rajalin & Fagerström, 2019; Vaartio‐Rajalin et al., 2019). Satisfied patients were also seen in the UK data as well and in both data sets HAH care was perceived to be economically effective and save hospital bed places: “If we did not exist, the patient would be an inpatient in the hospital.” In the UK, HAH care is free for patients at the point of access. HAH care in Finland is cheaper than hospital care. However, the structures and processes leading to these outcomes seem to be slightly different in Finland and the UK.

In both data sets, HAH care included the provision of specialized care to adult or older individuals with acute somatic health problems (c.f. Bäcklund et al., 2013) and also, only in Finland, the care of acute health problems linked to chronic somatic or mental health problems, as well as preventive, rehabilitative and palliative care. The referral process to HAH care appeared to be quite similar in both data sets, although in the UK the HAH staff were significantly greater in number and more interprofessional; in Finland, the HAH staff consisted of RNs and physicians (GPs or specialized physicians) who formed a team and thereafter consulted or collaborated with other professionals in accordance with patients' situations and needs (c.f. Larsen et al., 2017). Furthermore, in the UK the staff structure encompassed administrative staff and own (HAH) physiotherapists, pharmacists, occupational therapists, social workers and even drivers. Given the daily MDMs, one can assume that the communication and collaboration structures seen in the UK setting promoted patient care more systematically and more quickly than the interprofessional consultations seen in the Finnish setting. Moreover, during the daily MDMs seen in the UK, the separate documentation systems between professionals and units illuminated the goal of the meeting: the patient's best.

In both data sets, HAH staff's need for advanced clinical skills, garnered either through education, work experience or work rotation and which allowed the staff to iteratively see and analyse the whole situation for a patient both proactively and reactively and make independent decisions, was explicit. Differences seen between the two settings include that in the UK the GPs' main responsibility was to visit HAH patients in their homes while in Finland GPs more seldomly visit HAH patients. Also, HAH staff in the UK always make home visits in pairs and wear safety devices due to safety concerns, whereas in Finland home visits are usually conducted alone and without safety devices. One can therefore discern the huge demands placed on HAH staff's competence in Finland and the possible challenges associated with the work conditions in the Finnish setting. In the UK, matrons with a CNS or MNSC education played a central role in care planning and plan revisions (c.f. Fagerström, 2010; Öhlen et al., 2013). Yet in Finland only two participants were seen to possess an advanced education (in the form of ongoing APN education) and they performed the same tasks as the other Finnish HAH nurses, despite APNs being seen as central to the facilitation of patient‐centredness (Jeangsawang et al., 2012; Ljungbeck & Sjögren‐Forss, 2017; Pusa et al., 2015).

Nevertheless, it would seem that person‐centredness is more explicit in the Finnish rather than the UK setting in regard to HAH structures and processes. In Finland, both the HAH patient and his/her near‐ones are taken into consideration before and during HAH care and the pre‐admission phase was seen as balancing between the patient's and his/her near‐ones' opinions and wishes, while the HAH care process included focusing on both the patient and his/her near‐ones during care (c.f. Ewing et al., 2015; Farina, 2001; Landers et al., 2016; Vaartio‐Rajalin et al., 2019). This is in line with the definition of person‐centredness as the appropriate involvement of family and friends in decision‐making and information giving (c.f. Shaller, 2007) and respect for relationships and the recognition of this respect through the safeguarding of a partnership in shared decision‐making (c.f. Ekman et al., 2011; Kitwood, 1997; McCormack & McCance, 2006).

In Finland, the patient's home was perceived as balancing between the promotion of person‐centred care and own work safety, while in the UK work safety was paramount and better safety structures were in place, including working in pairs, own drivers and the use of safety devices, all for the purpose of ensuring time for the realization of person‐centred care. Both in Finland and the UK, verbal informed consent was sought from patients during the admission phase, but in Finland patients' near‐ones were also asked whether they accept HAH care and were ready to bear some responsibility as co‐clients (c.f. Vaartio‐Rajalin et al., 2019). Also, while in both settings patients were asked to provide feedback about HAH care, in the UK all HAH patients were given a health outcome form at the beginning and end of the care period and illness‐specific questionnaires (if available). This would be highly recommended also in Finland, because it would facilitate the gathering of evidence‐based data and thereby decision‐making in relation to the effectivity of and use of relevant resources in HAH.

Despite the slightly different patient groups, HAH in the UK setting appeared to have a stronger focus on tangible curative interventions and coordination than what was seen in the Finnish setting, which was “Coordinating and developing safe patient care through tangible and intangible measures.” This difference between the settings may be due to the continuous MDMs seen in the UK, where because each patient situation is discussed there is subsequently no need for additional, separate reports, documents or advocacy activities. It would be important to explore how such kinds of structures and processes affect HAH staff outcomes. As seen here, the HAH staff in Finland perceived they were continuously balancing between the patient's well‐being and his/her near‐one's integrity and balancing between a deeper meaning for one's work and the need for further support.

## 8 CONCLUSION

Based on the data sets seen here, it appears that HAH care in the UK is well structured and allows for processes and outcomes to be more easily identified than in HAH care in Finland. In Finland, where the HAH system is newer and still somewhat non‐systematic due to its vague structures and processes, a lot of balancing was seen between different extremes. Nonetheless, as only three units in Finland and one unit in the UK were included in this study, these results cannot be directly generalized.

## CONFLICT OF INTEREST

No conflicts of interest to state.
