# Supplementary material for: Balancing between extremes—Work in hospital‐at‐home
Source: Nurs Open. 2019 Oct 22;7(1):398–410. doi: 10.1002/nop2.402 (PMC6917957; doi:10.1002/nop2.402)
Supplement: Supplementary file 1 [file NOP2-7-398-s001.docx]

Consolidated criteria for reporting qualitative studies (COREQ): 32-item checklist

| **No** | **Item** | **Guide questions/description** |
| --- | --- | --- |
| **Domain 1: Research team and reflexivity** |  |  |
| Personal Characteristics |  |  |
| 1. | Interviewer/facilitator | Which author/s conducted the interview or focus group?  HVR (information sheet mentioned page 10) |
| 2. | Credentials | What were the researcher's credentials? *E.g. PhD, MD*  PhD (information sheet mentioned page 10) |
| 3. | Occupation | What was their occupation at the time of the study?  Researcher (information sheet mentioned page 10) |
| 4. | Gender | Was the researcher male or female?  Female (information sheet mentioned page 10) |
| 5. | Experience and training | What experience or training did the researcher have?  Several individual and focus-group interviews during PhD process and pots-doctoral research (information sheet mentioned page 10) |
| Relationship with participants |  |  |
| 6. | Relationship established | Was a relationship established prior to study commencement?  Research information sheet page 9 |
| 7. | Participant knowledge of the interviewer | What did the participants know about the researcher? e*.g. personal goals, reasons for doing the research*  Background, affiliations, reasons for doing the research, pages 9-10 |
| 8. | Interviewer characteristics | What characteristics were reported about the interviewer/facilitator? e.g. *Bias, assumptions, reasons and interests in the research topic*  None for there was no bias or assumptions, only research interest |
| **Domain 2: study design** |  |  |
| Theoretical framework |  |  |
| 9. | Methodological orientation and Theory | What methodological orientation was stated to underpin the study? *e.g. grounded theory, discourse analysis, ethnography, phenomenology, content analysis*  Content analysis, page 10 |
| Participant selection |  |  |
| 10. | Sampling | How were participants selected? *e.g. purposive, convenience, consecutive, snowball*  units with strategic sampling page 8-9 and respondents with convenience sample, page 8 |
| 11. | Method of approach | How were participants approached? e*.g. face-to-face, telephone, mail, email*  Research information sheet, page 9 |
| 12. | Sample size | How many participants were in the study? 24 + 1 in UK, pages 10-11 |
| 13. | Non-participation | How many people refused to participate or dropped out? Reasons?  7 nurses were not at work and therefore unable to participate. Not reported. |
| Setting |  |  |
| 14. | Setting of data collection | Where was the data collected? e*.g. home, clinic, workplace*  Workplace page 11 |
| 15. | Presence of non-participants | Was anyone else present besides the participants and researchers? No. Not reported. |
| 16. | Description of sample | What are the important characteristics of the sample? *e.g. demographic data, date* Working experience as a nurse, working experience at HAH, education; page 10 |
| Data collection |  |  |
| 17. | Interview guide | Were questions, prompts, guides provided by the authors? Was it pilot tested? Yes, page 9 and no |
| 18. | Repeat interviews | Were repeat interviews carried out? If yes, how many? No. Not reported. |
| 19. | Audio/visual recording | Did the research use audio or visual recording to collect the data? Yes, page 10 |
| 20. | Field notes | Were field notes made during and/or after the interview or focus group? Yes, page 10 |
| 21. | Duration | What was the duration of the interviews or focus group?  54-78 minutes, page 11 |
| 22. | Data saturation | Was data saturation discussed?  No. |
| 23. | Transcripts returned | Were transcripts returned to participants for comment and/or correction?  No |
| **Domain 3: analysis and findings**z |  |  |
| Data analysis |  |  |
| 24. | Number of data coders | How many data coders coded the data? One, and one checked. Not reported. |
| 25. | Description of the coding tree | Did authors provide a description of the coding tree? Yes, page 10 and Table 2 |
| 26. | Derivation of themes | Were themes identified in advance or derived from the data? Derived from the data, page 10 |
| 27. | Software | What software, if applicable, was used to manage the data? - |
| 28. | Participant checking | Did participants provide feedback on the findings? - |
| Reporting |  |  |
| 29. | Quotations presented | Were participant quotations presented to illustrate the themes / findings? Was each quotation identified? e*.g. participant number* Yes, by group code (under every citation) |
| 30. | Data and findings consistent | Was there consistency between the data presented and the findings? Yes, not reported |
| 31. | Clarity of major themes | Were major themes clearly presented in the findings? Yes, not reported |
| 32. | Clarity of minor themes | Is there a description of diverse cases or discussion of minor themes? No, for there were none. |
